# Clarification of the dispensability of *PDX1.2* for Arabidopsis viability using CRISPR/Cas9

**DOI:** 10.1186/s12870-019-2071-9

**Published:** 2019-11-04

**Authors:** Elisa Dell’Aglio, Ivan Dalvit, Sylvain Loubéry, Teresa B. Fitzpatrick

**Affiliations:** 1 Department of Botany and Plant Biology, University of Geneva, 1211 Geneva, Switzerland; 2 Present Address: Biologie Fonctionnelle, Insectes et Interactions, Institut National des Sciences Appliquées de Lyon, Institut National de la Recherche Agronomique, University of Lyon, F-69621 Villeurbanne, France

**Keywords:** Vitamin B_6_, CRISPR-Cas9, T-DNA insertion, Mutant alleles, Heat-stress, Heat shock element, *Arabidopsis thaliana*, PDX1.2, Viability

## Abstract

**Background:**

*PDX1.2* has recently been shown to be a regulator of vitamin B_6_ biosynthesis in plants and is implicated in biotic and abiotic stress resistance. *PDX1.2* expression is strongly and rapidly induced by heat stress. Interestingly, *PDX1.2* is restricted to eudicota, wherein it behaves as a non-catalytic pseudoenzyme and is suggested to provide an adaptive advantage to this clade. A first report on an Arabidopsis insertion mutant claims that *PDX1.2* is indispensable for viability, being essential for embryogenesis. However, a later study using an independent insertion allele suggests that knockout mutants of *pdx1.2* are viable. Therefore, the essentiality of *PDX1.2* for Arabidopsis viability is a matter of debate. Given the important implications of *PDX1.2* in stress responses, it is imperative to clarify if it is essential for plant viability.

**Results:**

We have studied the previously reported insertion alleles of *PDX1.2*, one of which is claimed to be essential for embryogenesis (*pdx1.2–1*), whereas the other is viable (*pdx1.2–2*). Our study shows that *pdx1.2–1* carries multiple T-DNA insertions, but the T-DNA insertion in *PDX1.2* is not responsible for the loss of embryogenesis. By contrast, the *pdx1.2–2* allele is an overexpressor of *PDX1.2* under standard growth conditions and not a null allele as previously reported. Nonetheless, upregulation of *PDX1.2* expression under heat stress is impaired in this mutant line. In wild type Arabidopsis, studies of PDX1.2-YFP fusion proteins show that the protein is enhanced under heat stress conditions. To clarify if *PDX1.2* is essential for Arabidopsis viability, we generated several independent mutant lines using the CRISPR-Cas9 gene editing technology. All of these lines are viable and behave similar to wild type under standard growth conditions. Reciprocal crosses of a subset of the CRISPR lines with *pdx1.2–1* recovers viability of the latter line and demonstrates that knocking out the functionality of PDX1.2 does not impair embryogenesis.

**Conclusions:**

Gene editing reveals that PDX1.2 is dispensable for Arabidopsis viability and resolves conflicting reports in the literature on its function.

## Background

The coenzyme pyridoxal 5′-phosphate (PLP) is essential for all organisms due to its involvement in numerous core metabolic enzyme reactions. PLP is biosynthesized de novo in plants, whereas animals must take it in their diet as vitamin B_6_. The pathway for biosynthesis of PLP in plants comprises just two enzymes PDX1 (PYRIDOXINE SYNTHASE1) and PDX2 (PYRIDOXINE SYNTHASE 2) [26–28]. These two enzymes form a complex that functions as a glutamine amidotransferase that utilizes ribose 5-phosphate, glyceraldehyde 3-phosphate and glutamine as substrates to facilitate PLP biosynthesis. There are three homologs of PDX1 in Arabidopsis (PDX1.1, PDX1.2 and PDX1.3), while there is only one homolog of PDX2 [27]. Of the PDX1s, only PDX1.1 and PDX1.3 are catalytic enzymes, whereas PDX1.2 is non-catalytic and is regarded as a pseudoenzyme. The term pseudoenzyme refers to the burgeoning family of proteins that are very similar to catalytic counterparts but do not function as enzymes [13]. In the case of PDX1.2, its classification as a pseudoenzyme refers to the fact that while it is expressed, key active site residues required for coordinating PLP biosynthesis are not conserved, rendering the protein catalytically non-functional [17]. The role of PDX1.2 appears to be as a positive regulator of PLP biosynthesis under environmental stress conditions, particularly under heat stress [4, 17]. Indeed, known homologs of *PDX1.2* from various plant species have a heat-shock element (HSE) in the region upstream of the translational start codon that binds heat shock transcription factors of the A1 family (HSFA1) [4]. Upregulation of *PDX1.2* abundance under heat stress appears to enhance stability of the catalytic PDX1s and thus sustain PLP levels under these conditions [4, 17], but the precise mechanistic details remain to be deciphered. In an attempt to provide insight into the nature of the interaction of PDX1.2 with its catalytic counterparts, we recently solved the X-ray crystal structures of complexes of PDX1.2 with PDX1.3 [23]. While the structure of the heteromeric PDX1.2-PDX1.3 complex is very similar to the structure of the PDX1.3 homomeric complex [22], we were unable to decipher key features that contribute to enhancement of PLP biosynthesis by PDX1.2, due to statistical disorder resulting from the near structural identity of both proteins. Nonetheless, recent studies of PDX1.2 have revealed a number of interesting features that include its restriction to eudicota and its important contribution to plant fitness [17]. Studies in which expression of *PDX1.2* was knocked down by RNA interference have rendered the plants more susceptible to disease, e.g. in tomato [32] and in Arabidopsis [31]; or more sensitive to abiotic stress, e.g. in Arabidopsis [17]. Interestingly, one study has reported that *PDX1.2* is essential for embryogenesis in Arabidopsis [14], based on the analysis of a T-DNA insertion mutant line of the SAIL collection [25]. However, a more recent study indicated that a null allele of *PDX1.2* was viable [31]. The study of additional mutant alleles of *PDX1.2* would help to clarify the effect of knocking out PDX1.2 functionality on Arabidopsis growth and development and resolve these contradictory reports. Furthermore, the study of pseudoenzymes has been neglected for a long time, due to their consideration as non-functional genes. However, pseudoenzymes are tightly conserved in sequence and although they have lost their catalytic capabilities, it is suggested that they have gained new functions [5]. A majority of those identified new functions are fulfilled by an allosteric control of bona fide enzymes (usually their catalytic homologs), e.g. in the case of kinases by either acting as a scaffold within a signaling cascade or by modulating the activity of gene expression modulators, such as transcription factors [10, 19]. Thus, although our knowledge of pseudoenzymes is still limited, they appear as regulators that can modulate physiological responses. In this context, it would be unexpected to find that a null mutation is embryonic lethal in a pseudoenzyme like PDX1.2, which is only induced in stress conditions. It is therefore important to clarify if PDX1.2 is essential for Arabidopsis viability.

While the large collections of Arabidopsis T-DNA insertion and EMS mutants have played a crucial role in investigations of gene function, the newly developed RNA-guided endonuclease-mediated targeted mutagenesis with the Clustered Regularly Interspersed Short Palindromic Repeats (CRISPR)/Cas9 methodology [11] facilitates the isolation of additional independent alleles. Having multiple mutant alleles to rigorously test hypotheses for gene functionality has been limiting in some previous studies. Indeed, the relative ease with which independent alleles can be generated with CRISPR-Cas9 technology is already proving to be important in the revision of original mechanistic hypothesis, e.g. the molecular function of the RIN protein in tomato [9]. Tomato *rin* mutants were isolated over half a century ago and were distinguished by an inability to ripen, lack of red pigmentation, as well as inability to soften or induce an ethylene burst [7], as is normally observed in climacteric fruit ripening [21]. Thus, RIN was assumed to be an activator, indispensable for induction of fruit ripening. Moreover, the discovery of *rin* led to this allele being integrated into food used for global consumption for decades. The recent isolation of CRISPR-Cas *rin* mutant alleles that ripen in the absence of RIN and associated studies demonstrate that the original *rin* mutant isolated is a gain of function mutant (not a loss of function mutant as previously assumed), which produces a protein that actively represses ripening, rather than activating it [9]. The study by Ito and colleagues [9] provides an excellent example of how the modern approach of gene editing technology can be used to improve our understanding of gene function and refine strategies for application of the findings.

Here we report on the use of CRISPR-Cas9 to clarify the notion that *PDX1.2* is essential for Arabidopsis viability. We studied both of the previously described T-DNA insertion mutants and generated several additional mutants of *PDX1.2* using CRISPR-Cas9. We report on the phenotype of loss of function mutants of *PDX1.2*, which clarify the dispensability of this gene under standard growth conditions and provide insight into *PDX1.2* transcriptional regulation. We also show that the PDX1.2 protein accumulates under heat stress conditions. These findings are important in the possible deployment of *PDX1.2* during applied studies in conferring tolerance to environmental stresses.

## Results

### Examination of *PDX1.2* mutant lines available from collections

**Fig. 1:**
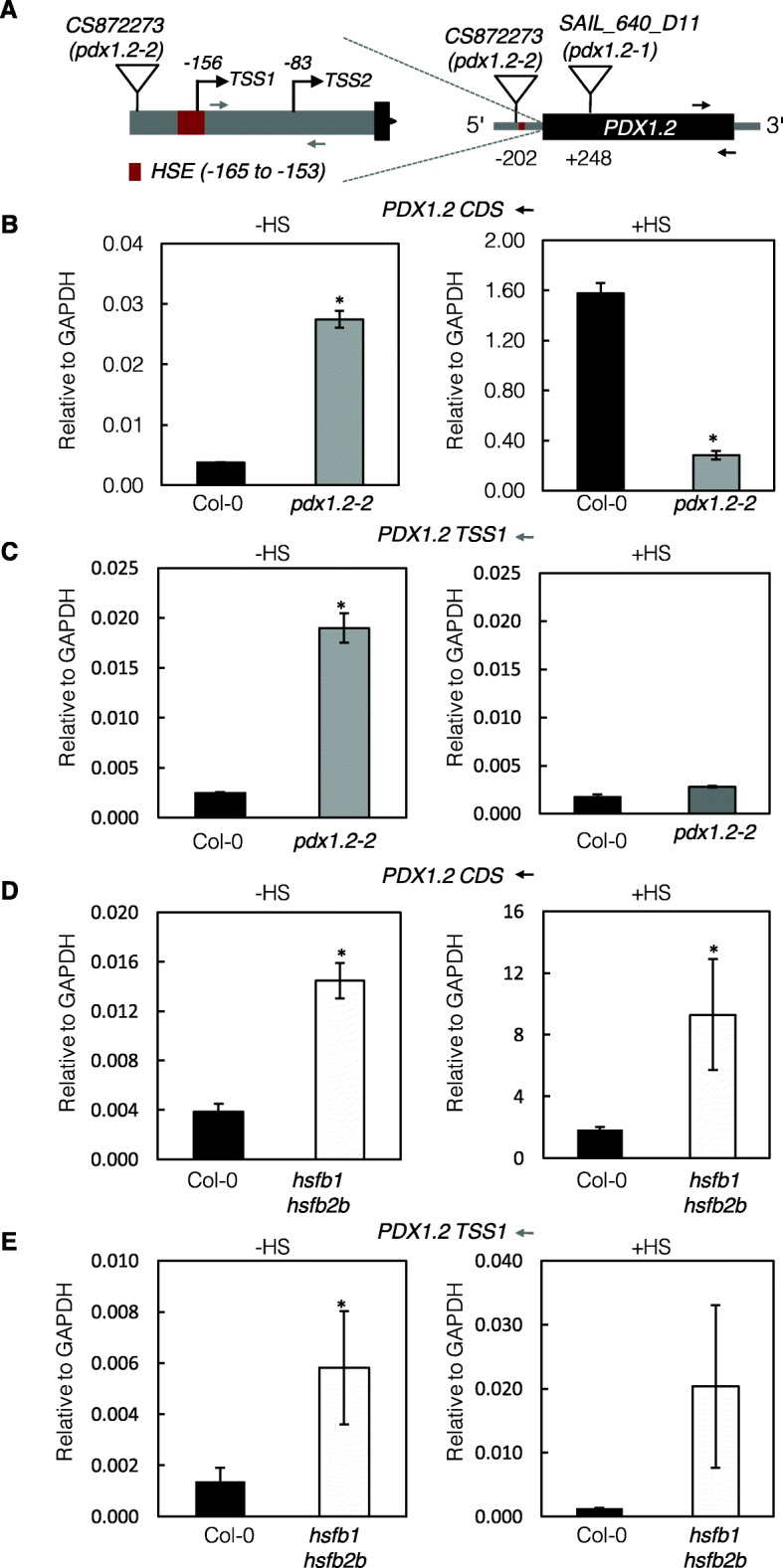
Characterization of *PDX1.2* expression in T-DNA insertion lines. **a** Schematic representation of *PDX1.2*. On the right, the single exon is depicted as a black box and the immediate upstream region is in gray and is further detailed on the left. The red box represents the heat shock element (HSE), which is from − 165 to − 153 bp upstream of the ATG translational start codon (+ 1). Alternative transcriptional start sites (TSS1 and TSS2) are indicated. The gray (TSS1) and black (protein coding sequence CDS)) arrow lines indicate the annealing positions of the primers used for qPCR. The locations of the T-DNA insertion in CS872273 (*pdx1.2–2*) and SAIL-640-D11 (*pdx1.2–1*) are as depicted and were confirmed by genotyping and sequencing. **b** and (**c**) Quantitative analysis of *PDX1*.*2* expression relative to GAPDH in *pdx1.2–2* compared to wild type (Col-0), in the absence (−HS) and presence of heat stress (+HS) using primers specific to the CDS or TSS1. Heat stress was induced by incubating seedlings for 1 h at 37 °C. **d** and (**e**) As for (**b**) and (**c**) but in *hsfb1 hsfb2b* compared to wild type (Col-0). Plants were grown in sterile culture under a 16-h photoperiod (120 μmol photons m^− 2^ s^− 1^) at 22 °C and 8 h of darkness at 18 °C. The data are the average of three biological and three technical replicates. Statistical differences from the wild type were calculated by a two-tailed Student’s *t* test and indicated by an asterisk for *P* < 0.001. In all cases, error bars represent SE

To define the requirement of *PDX1.2* expression for Arabidopsis viability, we obtained mutant lines from the available collections. Firstly, a segregating pool of seeds harboring the T-DNA insertion mutant line SAIL_640_D11 recently annotated as *pdx1.2–1* [14] was obtained from the European Arabidopsis Stock Centre. Molecular analysis indicated that a T-DNA insertion was within the coding region at position + 248 bp after the ATG translational start codon (Fig. 1a), corroborating the analysis reported by Leuendorf and colleagues [14]. As in the latter study, no lines homozygous for the T-DNA insertion could be found amongst the progeny of seeds. However, Southern blot analyses of this line using a probe matching a portion of the *BAR* gene, which forms part of the T-DNA construct, revealed that there are multiple T-DNA insertions (Additional file 1: Figure S1a,b). Notably, the sizes and number of the hybridizing restriction fragments, with several different restriction enzymes (Additional file 1: Figure S1a,b), are inconsistent with T-DNA insertions being at a single locus. Importantly, no hybridizing fragments could be detected with wild type DNA. Multiple T-DNA insertions in SAIL_640_D11 may explain the incongruities and inexplicable segregation outcomes with respect to *PDX1.2* reported previously [14]. The latter study did not perform a Southern analysis or sequence the genome of this line annotated as *pdx1.2–1*.

A second T-DNA insertion line, CS872273, which we have annotated here as *pdx1.2–2* was also obtained from the European Arabidopsis Stock Center. Our molecular analysis revealed that the T-DNA insertion was located in the promoter region of *PDX1.2* at position − 202 bp before the ATG translational start codon (Fig. 1a). Seeds homozygous for the T-DNA insertion could be isolated from segregating progeny. This mutant line has previously been reported as a null mutant allele for *PDX1.2* expression but in stark contrast to *pdx1.2–1* is reported to be viable and indistinguishable from wild type under standard growth conditions [31]. Indeed in our hands, growth of *pdx1.2–2* under a 16-h photoperiod at 22 °C, 8 h of darkness at 18 °C, (ambient CO_2_, 60% relative humidity) did not distinguish it from wild type plants grown under the same conditions (hereafter referred to as standard conditions). However, in contrast to the study of Zhang and colleagues [31], our quantitative real-time RT-PCR (qPCR) analysis of *pdx1.2–2* grown under standard conditions indicated increased expression of *PDX1.2* in this line compared to wild type (Fig. 1b, left panel). Expression of *PDX1.2* has recently been shown to be transcriptionally upregulated by heat stress [17] and is mediated by the HSFA1 transcription factor family [4]. Interestingly, here we observed that induction of *PDX1.2* by heat stress was considerably attenuated in *pdx1.2–2* compared to wild type under the same conditions (Fig. 1b, right panel).

In the context of the above observations, we noted that the T-DNA insertion in *pdx1.2–2* (at − 202 bp relative to the ATG translational start site) is just upstream of the HSE (at − 165 to − 153 bp relative to the ATG translational start site) in the promoter region of *PDX1.2* (Fig. 1a). It has been shown previously that there are two transcriptional start sites (TSS1 and TSS2) in the region immediately upstream of the translational start site in *PDX1.2* [4] at bp − 156 and bp − 83, respectively (Fig. 1a). TSS1 is within the HSE to which HSFA1 binds to mediate induction of *PDX1.2* expression under heat stress conditions. Consequently, TSS2 that is downstream of the HSE is used as an additional or alternative transcriptional start site under these conditions [4]. To provide more insight into the observations with the *pdx1.2–2* mutant, we quantified the level of transcripts starting from TSS1 (using a primer pair that anneals either side of TSS2 (Fig. 1a)) in the absence and presence of heat stress. *PDX1.2* transcript levels as a function of TSS1 were similar under both conditions in wild type (Fig. 1c), i.e. increased transcript abundance under heat stress is predominantly derived from TSS2 [4]. By contrast, enhanced expression observed in the absence of heat stress in *pdx1.2–2* was considerably attenuated in the presence of heat-stress and in fact similar to wild-type transcript levels as a function of TSS1 (Fig. 1c). In this context, it is interesting to know that HSFB1 and HSFB2b are suppressors of heat stress induced genes [8] and it was previously noted that *PDX1.2* transcript levels are enhanced in a global transcriptome analysis of the *hsfb1 hsfb2b* double mutant under standard growth conditions [4, 8]. Indeed, we could confirm increased expression of *PDX1.2* in the absence of heat stress and show that overall expression of *PDX1.2* was enhanced in the presence of heat stress in *hsfb1 hsfb2b* compared to wild type (Fig. 1d). Moreover, quantification of transcripts derived from the use of TSS1 in *PDX1.2* in the presence and absence of heat stress, show that while they remain the same in the wild type, they are increased under heat stress in the *hsfb1 hsfb2b* mutant (Fig. 1e). This observation is consistent with the hypothesis that HSFA1 and HBFB1/2b regulate expression of *PDX1.2* probably through the HSE. We postulate that the close proximity of the T-DNA insertion to the HSE in *pdx1.2–2* interferes with the binding of HSFB1/2b and HSFA1 and therefore contributes to the transcriptional increase under standard growth conditions and to the transcriptional attenuation observed under heat stress conditions and could be studied in more elaborative studies of the heat stress response in the future.

Taken together, we conclude that *pdx1.2–2* can be considered as an overexpression mutant under standard growth conditions and an underexpression mutant under heat stress conditions.

### PDX1.2 protein levels are induced by heat stress

**Fig. 2:**
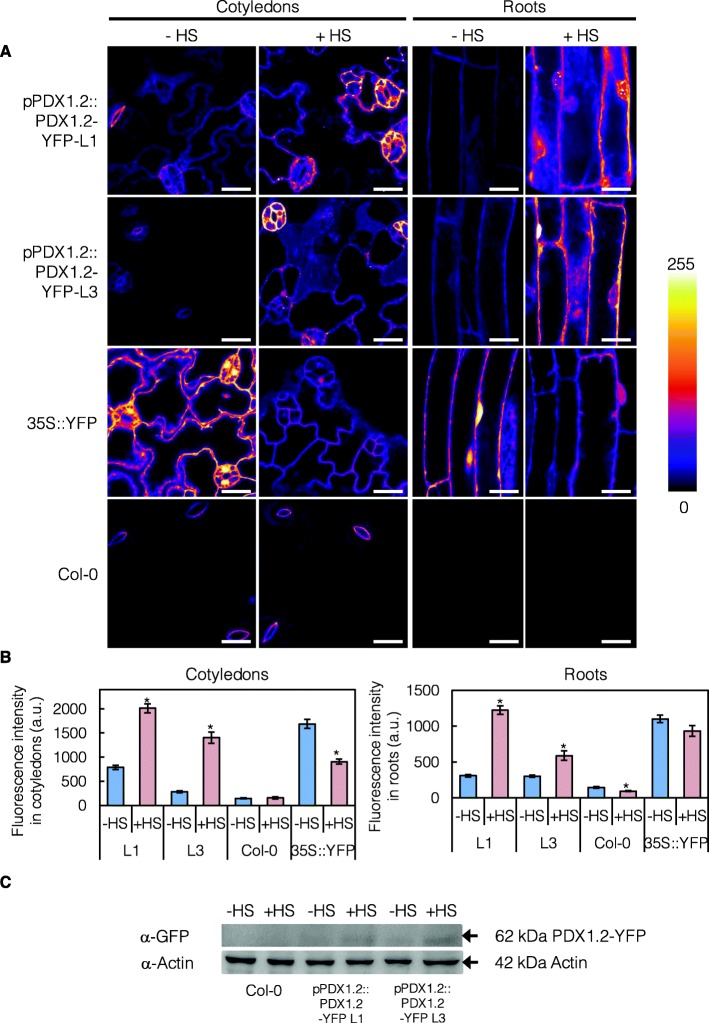
The PDX1.2 protein accumulates upon heat stress. **a** Confocal micrographs (z slices) of cotyledons and roots of 8-days-old *Arabidopsis* expressing the PDX1.2-YFP fusion protein under the control of the upstream region of PDX1.2 (pPDX1.2::PDX1.2-YFP), in the absence (−HS) and presence of heat stress (+HS). L1 and L3 refer to independent lines. Heat stress was induced by incubating seedlings for 3 h at 37 °C. YFP expressed alone under the control of the *CaMV 35S* promoter (35S::YFP) and non-transgenic wild type (Col-0) are also shown for comparison. Scale bars: 20 μm. A color scale bar of fluorescence intensity is shown on the right. **b** Fluorescence intensities (arbitrary units) measured in cotyledons and in roots. Note that 35S-YFP plants were imaged with different acquisition parameters than the other lines, making the absolute values measured in this line not comparable with those measured in the others. The data are the average of 8–67 tissues from at least 2 plants per genotype, tissue and condition (see methods) and are represented as the average ± SE. Statistical differences were calculated by a two-tailed Student’s *t* test for genotype/tissue with and without heat stress and are indicated by an asterisk for *P* < 0.05. **c** Immunochemical analysis of 8-days-old whole seedlings of independent pPDX1.2::PDX1.2-YFP lines (L1 and L3) compared to wild type (Col-0) using an antibody against GFP (α-GFP). An antibody against actin (α-Actin) was used as a loading control. The arrows point to labelled bands at 62 kDa and 42 kDa, the expected sizes of the PDX1.2-YFP fusion protein and actin, respectively. Samples correspond to treatment with heat stress (+HS) or non-treatment (−HS) as shown for part (**a**)

Although we have previously shown that *PDX1.2* expression is upregulated by heat stress at the transcript level and corroborate the observation here in this study, direct evidence for accumulation of the protein under these conditions has not been provided. To assess accumulation of the protein under heat stress, we constructed a translational fusion of PDX1.2 with YFP under the control of the upstream region (− 1 to − 1495 bp upstream of the translational start codon) of *PDX1.2.* Lines stably expressing the fusion protein were generated and annotated pPDX1.2::PDX1.2-YFP. The fluorescence of pPDX1.2::PDX1.2-YFP lines was monitored in cotyledons and in root epidermal cells and was above that observed in non-transformed control lines, although lower than that observed in the control 35S-YFP line in the absence of heat stress (Fig. 2a, see – HS panels). At the subcellular level, the PDX1.2-YFP fusion proteins were predominantly localized to the cytosol but sometimes also found in small foci (Fig. 2a, see – HS panels). This corroborates earlier observations upon transient expression of PDX1.2-GFP in Arabidopsis mesophyll protoplasts [27]. Interestingly, exposure of pPDX1.2::PDX1.2-YFP lines to heat stress (+ HS) enhanced the level of protein based on fluorescence intensity, whereas the level was reduced in the control 35S-YFP line (Fig. 2b, compare ± HS). Notably, a reduction of translation and/or ribosome stalling under heat stress conditions is characteristic of most proteins particularly those not involved in the heat shock response [30]. The enhanced level of fluorescence upon heat stress with pPDX1.2::PDX1.2-YFP was particularly concentrated in the guard cells, at least in cotyledons (Fig. 2a). The enhanced expression of PDX1.2-YFP under heat stress was confirmed by immunodetection of the protein using an antibody directed against the fluorescent protein (Fig. 2c).

### Generation of *PDX1.2* mutant lines by CRISPR-Cas9 activity

**Fig. 3:**
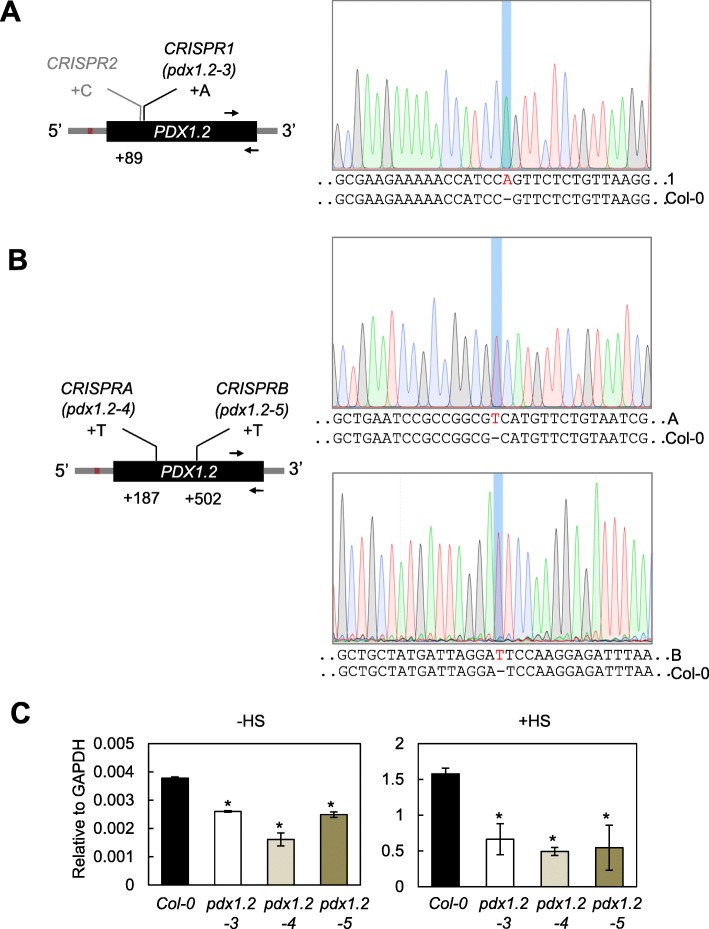
Generation of independent alleles of *pdx1.2* by CRISPR-Cas9. **a** and (**b**) Schematic representations of alleles of *pdx1.2* generated using the CRISPR-Cas9 technology are shown on the left. The red box represents the heat shock element (HSE), which is from − 165 to − 153 bp upstream of the ATG translational start codon (+ 1). The black arrows indicate the annealing positions of the primers used for qPCR. The numbers refer to the site of insertion of a nucleotide as depicted. DNA sequencing chromatograms around the mutated sites are shown on the right. The DNA sequences of wild type (Col-0) and CRISPR mutations (1 (*pdx1.2–3*), A-1.8 (*pdx1.2–4*), B-11.11 (*pdx1.2–5*)) are given below each chromatogram. In each case, CRISPR resulted in the addition of a nucleotide, as depicted (in red), and is highlighted by a blue bar in the respective chromatogram. **c** Quantitative analysis of *PDX1*.*2* transcript expression in wild type (Col-0) and the *pdx1.2* CRISPR mutants characterized (*pdx1.2–3*, *pdx1.2–4*, *pdx1.2–5*). The expression relative to GAPDH in the respective lines is depicted in the absence of heat stress (-HS) or the presence of heat stress (+HS). Heat stress was induced by exposure to 37 °C for 1 h, at which time-point samples were harvested. In each case, 8-days-old Arabidopsis seedlings pre-cultivated in sterile culture at 22 °C under a 16-h photoperiod (120 μmol photons m^− 2^ s^− 1^) and 8 h of darkness at 18 °C were used. The data are the average of three biological and three technical replicates. Statistical differences from the wild type under the same conditions were calculated by a two-tailed Student’s *t* test and indicated by an asterisk for *P* < 0.001. In all cases, error bars represent SE

To clarify the dispensability of *PDX1.2* for Arabidopsis viability and as an independent approach to generate null alleles of *PDX1.2* we used RNA-guided endonuclease-mediated targeted mutagenesis with the Clustered Regularly Interspersed Short Palindromic Repeats (CRISPR)-Cas9 system (Li et al., 2013). In the first instance, we employed the non-homologous end-joining (NHEJ) method described by Fauser et al. [6] using the plasmids pEN-Chimera and pDE-Cas9. We used an sgRNA that targeted the N terminal end of *PDX1.2*, the region most divergent to the catalytic PDX1.1 or PDX1.3, and after selection of transformants could identify two lines harboring *PDX1.2* mutations (CRISPR1 and CRISPR2) in the C_2_ generation. However, only CRISPR1 could be confirmed as a single insertion, Cas9-free and homozygous in the C_3_ generation. CRISPR2 was likely to be a chimera or heteroallelic and was not pursued further. CRISPR1 carries an A insertion at + 89 bp and was annotated *pdx1.2–3* (Fig. 3a). Subsequently, due to the progression of the technology, we generated mutant alleles of *PDX1.2* with the method described by Wang et al. [29] using the plasmids pHEE2A-TR1 and pHEE-401E. The latter targets egg cells (employing the *EC1.2* gene promoter) and thus reduces or eliminates the mosaicity observed with the previous approach and potentially facilitates the isolation of biallelic mutants in one generation [29]. We used four sgRNAs in two different constructs (see methods) and obtained one heterozygous mutant for each in the C_1_ generation, which were subsequently isolated to homozygosity. Sequencing of the region surrounding the target sites confirmed that they were mutants and harbored insertion of a T at either + 187 bp (*pdx1.2–4*, CRISPRA) or + 502 bp (*pdx1.2–5*, CRISPRB), respectively (Fig. 3b). In all of the transgenic CRISPR lines, a premature stop codon is generated after the respective single base pair insertion due to a frame shift, which leads to truncated versions of PDX1.2. The largest of these truncated versions that could be generated, if transcribed in frame, is with CRISPRA, which could lead to a shorter version of PDX1.2 that would be 233 amino acids (i.e. missing 81 amino acids from the N terminus). Full-length PDX1.2 alone is expected to fold as a β/α_8_ barrel similar to its paralogs [22, 26]. Moreover, this fold is necessary for the observed interaction with catalytic PDX1 counterparts (e.g. PDX1.3) and to confer functionality within a PDX1.2/catalytic PDX1 protein complex [17]. Our recent X-ray crystal structure data of the PDX1.2-PDX1.3 heteromeric complex confirms these previous hypotheses and shows that PDX1.2 folds as a β/α_8_ barrel [23]. Even the largest truncated version of PDX1.2 that could be generated in any of the CRISPR mutants would be unable to assemble into this fold. Therefore, the CRISPR mutants *pdx1.2–3*, *pdx1.3–4* and *pdx1.2–5* are expected to be non-functional. Furthermore, we measured the overall *PDX1.2* transcript levels in the generated mutants and observed that they were lower (i.e. in *pdx1.2–3*, *pdx1.3–4* and *pdx1.2–5*) under standard growth conditions, as well as under heat stress conditions compared to wild type (Fig. 3c). This may be explained if the single nucleotide insertion in these respective mutants leads to a less stable version of the transcripts under these conditions. Future work will establish if this is the case.

### Phenotypic analyses of *PDX1.2* mutant lines

**Fig. 4:**
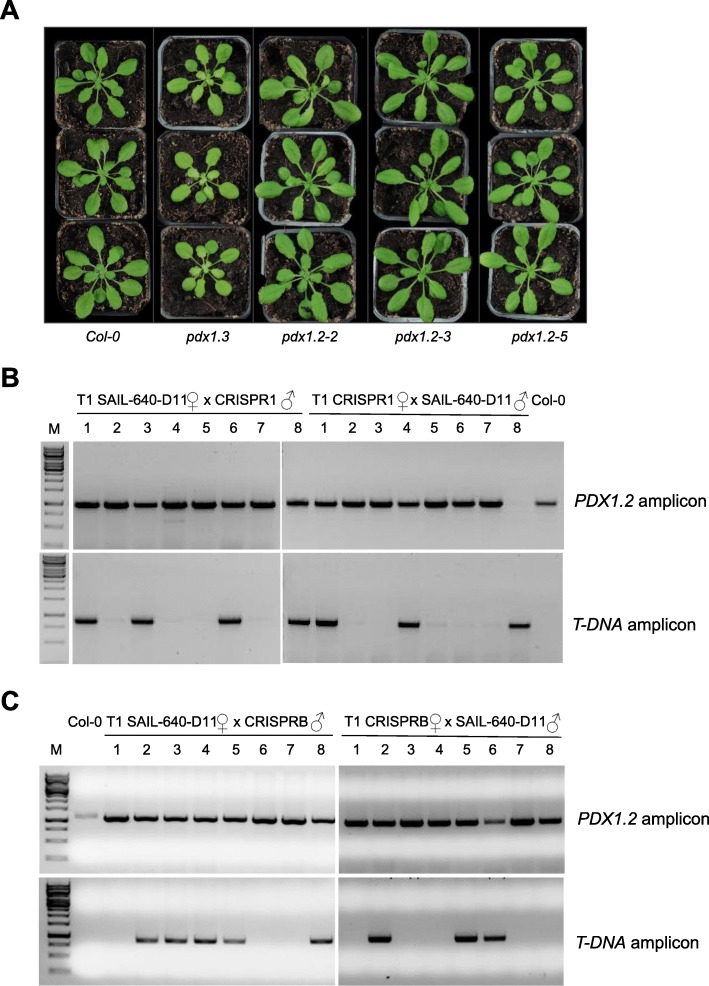
*PDX1.2* is dispensable for Arabidopsis development under standard conditions. **a** Photographs of *PDX1* lines as indicated grown under an 8 h photoperiod (120 μmol photons m^− 2^ s^− 1^) at 22 °C and 16 h of darkness at 18 °C for 27 days after germination, compared to wild-type. **b** and (**c**) Genotyping of reciprocal test crosses of either CRISPR1 (*pdx1.2–3*) (**b**) or CRISPRB (*pdx1.2–5*) (**c**) with SAIL-640-D11 (*pdx1.2–1*) for the T-DNA amplicon in *pdx1.2–1* and a *PDX1.2* amplicon in the T_1_ generation. Representative analyses of eight plants from each cross are shown compared to wild type (Col-0). Both amplicons can be found in approximately 50% of the plants. DNA sizing ladder is shown in the lane marked M

Given the contrasting reports in the literature with regard to the phenotype of loss of function *PDX1.2* mutant lines [14, 31], we combined the newly created CRISPR mutants in a (re) analysis for growth impairment under our standard growth conditions. As demonstrated above the mutants *pdx1.2–3*, *pdx1.2–4* and *pdx1.2–5* are considered loss of function mutants, whereas *pdx1.2–2* is enhanced in *PDX1.2* expression under these conditions. Either under a 16-h or 8-h photoperiod at an ambient temperature of 22 °C during the light period, no incongruent phenotype that would distinguish the *pdx1.2* mutant lines (with the exception of *pdx1.2–1*, which is embryo lethal and not included here) from wild type could be discerned (see Fig. 4a for a representative example of growth phenotypes). This corroborates the previous report with respect to *pdx1.2–2*, but contradicts the notion that *pdx1.2* is essential for embryogenesis [14]. Notably, we used a previously described mutant of *pdx1.3* [28] as a control and it was seen to display chlorosis of newly emerging leaves and stunted growth, the typically reported phenotype of this mutant under these conditions [28] (Fig. 4a).

*PDX1.2* has been reported to be critical for embryo development based on an analysis of a single T-DNA insertion mutant line SAIL_640_D11 (*pdx1.2–1*) [14]. In this report, it was not clear if the observed phenotype was a combination of gametophytic and embryogenesis impairment or solely the latter due to non-conforming segregation ratios. Given the results of our Southern blot analysis, we decided to further probe SAIL_640_D11 as a function of *PDX1.2*. In the first instance, based on the assumption that the mutant *pdx1.2–3* generated in this study is a loss of function mutant (i.e. truncated protein missing the N terminus and unlikely to fold properly), we performed reciprocal crosses of heterozygous *pdx1.2–1* (*PDX1.2 pdx1.2–1*) with *pdx1.2–3* (CRISPR1). If *PDX1.2* is indeed critical for embryo development as reported and *pdx1.2–3* is non-functional, then we would not expect to find *pdx1.2–1 pdx1.2–3* in the F_1_ generation. On the other hand, if *pdx1.2–3* can overcome the mutation in *pdx1.2–1* then the progeny, analyzed at the seedling stage, should segregate 1:1 for *pdx1.2–1 pdx1.2–3* and *PDX1.2 pdx1.2–3*. A segregation analysis of *PDX1.2* gene and T-DNA insertion specific PCR of F_1_ seedlings derived from this cross demonstrated that approximately half of the progeny carry the T-DNA insertion of *pdx1.2–1* (Fig. 4b). Thus, we can conclude that *pdx1.2–3* can overcome the mutation in *pdx1.2–1*. If *pdx1.2–3* is indeed non-functional, then loss of PDX1.2 is not critical for embryogenesis.

To probe this further, we performed a similar analysis using another of the CRISPR mutants generated, *pdx1.2–5*. The premature stop codon in the *pdx1.2–5* mutant leads to a protein that is estimated to be only half the size (19.2 kDa if translated from the first ATG and/or 13.6 kDa if a product is translated from an ATG after the premature stop codon) of the mature protein (33.8 kDa) and cannot be functional based on the necessity to fold as a β/α_8_ barrel [17, 23]. Reciprocal crosses of heterozygous *pdx1.2–1* (*PDX1.2 pdx1.2–1*) with *pdx1.2–5* (CRISPRB) and a segregation analysis of *PDX1.2* gene and T-DNA insertion specific PCR of F_1_ seedlings derived from this cross showed that approximately half of the progeny carry the T-DNA insertion of *pdx1.2–1* (Fig. 4c). Therefore, *pdx1.2–5* (as for *pdx1.2–3*) can overcome the mutation in *pdx1.2–1*.

Taken together, we conclude that loss of PDX1.2 is not critical for embryogenesis in Arabidopsis.

## Discussion

The CRISPR-Cas system that originated from prokaryotes as an adaptive immunity tool has been exploited extensively in the last few years as a precision tool to achieve genome editing. The technology is powerful in that several independent mutations can be generated relatively quickly in most organisms. Here we used the system to generate mutants of *PDX1.2*, as previous reports on phenotypes of T-DNA insertion mutant lines were inconsistent. The first T-DNA insertion mutant line (*pdx1.2–1*) studied, led to the claim that *PDX1.2* is necessary for embryogenesis, as the *pdx1.2–1* mutant was unviable [14]. However, from our study here, it is clear that there are several T-DNA insertions in this line, which would explain the previously reported incongruities and inexplicable segregation outcomes with respect to *PDX1.2* [14]. Moreover, we have shown here that crossing the *pdx1.2–1* line with independent CRISPR mutants of non-functional *PDX1.2*, allows us to isolate mutants that carry both the T-DNA insertion from *pdx1.2–1* and the *PDX1.2* CRISPR mutations. Therefore, these crossed lines effectively rescue the embryogenic defect and show that *PDX1.2* is dispensable for growth under the conditions used (i.e. standard laboratory conditions). On the other hand, the T-DNA insertion mutant line, *pdx1.2–2*, was previously described as a loss of function line or null allele [31]. The latter study did not report the location of the T-DNA insertion. However, our analyses indicate that the insertion is at − 202 bp upstream of the ATG translational start site. This places the T-DNA insertion just upstream of the validated HSE (at − 165 to − 153 bp relative to the ATG translational start site) in the *PDX1.2* promoter region [4]. This is relevant because although a strong induction of *PDX1.2* expression is observed under heat stress in wild type plants and is under control of the HSFA1 transcription factor family, we previously hypothesized that expression may be actively repressed under ambient conditions [4]. The latter hypothesis stemmed from the observation that *PDX1.2* is among the set of genes induced under ambient conditions in a global transcriptome analysis of the *hsfb1 hsfb2b* double mutant [8]. Here, we provide supporting evidence for this hypothesis by specifically examining the response of *PDX1.2* expression in the absence and presence of heat stress in *hsfb1 hsfb2b* compared to wild type (Fig. 1d). The HSFB1 and HSFB2b heat shock factors suppress expression of heat-shock inducible genes under ambient conditions and are thought to mediate their function through the HSE-like consensus sequences [8]. Indeed, the *hsfb1 hsfb2b* mutant is considered to be in a constitutive *moderate* heat stress state, with several heat shock response genes induced in this mutant [8]. Thus, given the location of the T-DNA insertion in *pdx1.2–2,* i.e. relatively close to the HSE, and the enhanced expression of *PDX1.2* in *pdx1.2–2* under ambient conditions, we are tempted to speculate that the suppression of expression is impaired in this mutant, i.e. explaining enhanced expression of *PDX1.2* under ambient conditions in this mutant. In addition, the T-DNA insertion may interfere with HSFA1 binding to the HSE under heat stress conditions, which would account for the observed attenuation of the response in *pdx1.2–2* compared to wild type under these conditions.

The analysis of PDX1.2-YFP fusion proteins corroborate the previous hypothesis that PDX1.2 is upregulated by heat stress and may serve to stabilize its catalytic counterparts, i.e. PDX1.1 or PDX1.3 in Arabidopsis [4]. Indeed, we have recently been able to solve the crystal structure of the complex of PDX1.2 with PDX1.3 [23]. Unfortunately, statistical disorder prevented us from distinguishing PDX1.2 from PDX1.3 in the complex. Nevertheless, we could conclude that although PDX1.2 has very subtle effects on the conformation of PDX1.3, it likely serves to prime key catalytic regions for functionality in vitamin B_6_ biosynthesis. It is interesting that PDX1.2 protein accumulation is observed in the guard cells under heat stress conditions. It is well established that one of the first physiological responses of Arabidopsis (and many other plants) to heat stress takes place at the guard cells. Thus, this may be the site where PDX1.2 action is required most during such stress conditions.

## Conclusions

In this study, we have examined several mutant alleles of *PDX1.2* with the aim of defining the dispensability of this gene under standard laboratory growth conditions for Arabidopsis viability. Specifically, we characterized the previously reported null alleles of *PDX1.2* and generated additional null alleles using the CRISPR-Cas9 technology. Our studies show that *PDX1.2* is dispensable for growth under normal conditions and its absence does not induce embryo lethality as previously reported. Moreover, one of the previously reported *pdx1.2* null alleles is in fact enhanced in *PDX1.2* expression under standard conditions but attenuated under heat stress conditions, likely due to misregulation in the vicinity of the HSE involving the transcription factors HSFA1 and HSFB1/2b. Our data reinforces the fact that definition of gene functionality requires the rigorous analysis of multiple alleles and that consistent outcomes as well as conclusions can be readily achieved in this era by making use of current gene editing techniques. Indeed, this study provides a good example of the use of CRISPR-Cas to resolve issues related to conflicting reports on the functionality of particular genes.

## Methods

### Plant material and growth conditions

*Arabidopsis thaliana* (Columbia-0 ecotype) was used throughout. The T-DNA insertion mutant lines SAIL_640_D11 [25] and CS872273 [31] annotated as *pdx1.2–1* in [14] and *pdx1.2–2* in this study, respectively, were obtained from the European Arabidopsis Stock Center (NASC). The *hsfb1–1 hsfb2b-1* (*hsfb1 hsfb2b*) seeds were a generous gift from Masaru Ohme-Takagi, National Institute of Advanced Industrial Science and Technology, Japan. Seeds cultivated in sterile culture were surface-sterilized in 70% ethanol (v/v) and dried prior to plating on half-strength MS medium without vitamins [18] (Duchefa, http://www.duchefa.com) containing 0.8% agar (w/v) in Petri dishes. Seeds cultivated under non-sterile conditions were sown on soil (Einheitserde, Classic Ton Kokos). Seeds were stratified for two to four days at 4 °C in the dark before transfer to a growth incubator (CLF Climatics CU-22 L for sterile cultures; CLF Climatics AR-66 for soil grown cultures). Plants were grown either under long-day (16 h) or short day (8 h) photoperiods (100 to 150 μmol photons m^− 2^ s^− 1^generated by fluorescent lamps [Philips Master T-D Super 80 18 W/180]) at 22 °C and 60% relative humidity, followed by either 8 h or 16 h of darkness at 18 °C, respectively, all at ambient CO_2_. Eight-day-old seedlings grown under long-day photoperiods were used for heat stress experiments. Heat stress at 37 °C was achieved by transferring the seedlings to an incubator (CLF Climatics I-30Bl4/D) at the defined temperature with the remaining conditions as above (100–150 μmol photons m^2^ s^− 1^, 60% relative humidity and ambient CO_2_) for 1–3 h, as indicated. Plant lines carrying the *pdx1.2–1* and *pdx1.2–2* T-DNA insertions were verified by PCR analysis of genomic DNA (see Additional file 2: Table S1 for oligonucleotides used). The level of expression of *PDX1.2* in the respective lines was verified by qPCR (see below). Plants homozygous for either *pdx1.2–3* (CRISPR1) or *pdx1.2–5* (CRISPRB) were crossed with those heterozygous for *pdx1.2–1* (*PDX1.2/pdx1.2–1*). The F_1_ progeny were analyzed, which are heterozygous for either *pdx1.2–3* or *pdx1.2–5* and *PDX1.2/pdx1.2–1*.

### Molecular methods

Southern blot analysis was carried out using genomic DNA isolated from leaves of heterozygous lines of *pdx1.2–1* (*PDX1.2 pdx1.2–1*) and the corresponding wild type (Col-0) plants, grown under long-day conditions. DNA samples (15 mg) were digested overnight at 37 °C with either the HindIII, EcoR1, SacI or NcoI (150 U) restriction enzyme, as indicated, in a final volume of 75 μl followed by electrophoresis on a 0.7% agarose gel. The digested samples were probed with an anti-digoxigenin (DIG) labeled fragment matching a portion of the *BAR* gene, which forms part of the T-DNA construct used to produce the SAIL collection [25]. The probe was prepared via PCR amplification from the pDAP101 plasmid using primers CGAAATAAACGACCAAATTAGTAGAA and ACCCTATAAGAACCCTAATTCCCTTAT (Additional file 2: Table S1). The probe DNA was labeled using the PCR DIG probe synthesis mix (Sigma-Aldrich), hybridized overnight in DIG EasyHyb buffer containing DIG-AP Fab fragments (Roche) against neutrally-charged nylon membranes (0.45 μm pore size, Nytran). Membranes were prepared for imaging with the DIG luminescence detection kit (Sigma-Aldrich), before exposure against Super RX X-ray film (Fujifilm).

For gene expression analyses by real-time quantitative reverse transcription PCR (qPCR), tissue samples were collected from 8-day-old seedlings grown under long-day conditions. RNA was extracted using the PureLink RNA Mini kit (Ambion) according to the manufacturer’s instructions. DNA was removed by an on-column DNase digest during the RNA extraction. Reverse transcription was performed using 0.5 μg total RNA as a template, Superscript II reverse transcriptase (200 U) and oligo (dT)_20_ primers (500 ng) (Thermo Fisher Scientific). qPCR was performed in 384 well plates on a 7900HT Fast Real-Time PCR system (Applied Biosystems) using Power SYBR Green master mix (Applied Biosystems) and the following amplification program: 10 min denaturation at 95 °C followed by 40 cycles of 95 °C for 15 s and 60 °C for 1 min. The data were analyzed using the comparative cycle threshold method (2^−ΔCT^) normalized to the Arabidopsis reference gene *GAPDH* (At1g13440). Each experiment was performed with three biological and three technical replicates. In all cases, primer pairs used are given in Additional file 2: Table S1.

### Generation of PDX1.2-YFP lines, molecular analysis and confocal microscopy

For expression of *PDX1.2-YFP*, *PDX1.2* without its stop codon was amplified from cDNA of 8-days-old seedlings using Phusion proofreading polymerase (ThermoFisher) and specific primer pairs (PDX1.2-YFP F, GGGGACAAGTTTGTACAAAAAAGCAGGCTATGGCGGATCAAGCTATGAC and PDX1.2-YFP R, GGGGACCACTTTGTACAAGAAAGCTGGGTAACACTGCCTTGGCCAAAGTC). The amplified products were purified and cloned into the pDONR221 vector by the BP recombination reaction using BP Clonase™ II mix (ThermoFisher) according to the manufacturer’s instructions to generate pDONR221:PDX1.2-YFP, sequenced, and subsequently cloned into the destination vector pB7YWG2 [12] by an LR reaction using the LR Clonase™ mix II (ThermoFisher) to generate pB7YWG2::PDX1.2-YFP. Afterwards, the region comprising bp − 1 to − 1495 upstream of the ATG translational start codon of PDX1.2 was amplified from genomic DNA of 8-days-old seedlings using the primer pair pPDX1.2::PDX1.2-YFP F, AATAT*GAGCTC*TTAATTATCTCTCTCAATGAG and pPDX1.2::PDX1.2-YFP R, ATATTA*ACTAGT*TTTTAGGTTCTGTGAGTTTTTAGTAACAG, where the regions in italics denote implemented SacI and SpeI restriction sites, respectively. The amplicon was digested with SacI and SpeI, purified and ligated into similarly digested and purified pB7YWG2 to replace the *CaMV 35S* promoter to generate pPDX1.2::pB7YWG2. Subsequently, pDONR221::PDX1.2-YFP and pPDX1.2::pB7YWG2 were recombined by an LR reaction using the LR Clonase™ mix II (ThermoFisher) to generate pB7YWG2::pPDX1.2::PDX1.2-YFP. In this case, PDX1.2 will be expressed as a fusion protein with YFP at the C terminus. The construct and the empty vector (pB7YWG2) as a control were introduced into *Agrobacterium tumefaciens* strain C58 and used to transform wild type (Col-0) *Arabidopsis* plants by the floral dip method [2]. As the respective constructs contain the *BAR* gene, transformants were selected by resistance to BASTA™. Resistant plants were allowed to self-fertilize, and homozygous lines were selected from the T_3_ generation according to their segregation ratio for BASTA™ resistance. Eight-days-old transgenic seedlings grown in sterile culture under long-day conditions, as described above were used for confocal microscopy. The heat stress treatment was performed by exposing seedlings to 37 °C for 3 h before microscopic analysis. In each case, seedlings were mounted in water between the slide and the coverslip with a double-sided Scotch tape spacer. They were imaged with an SP5 confocal laser-scanning microscope (Leica) equipped with a resonant scanner and a × 40 Oil, numerical aperture 1.25 PlanApo lens. The pinhole was set at 1 Airy unit and the zoom was set so that the pixel size was between 140 and 150 nm. YFP was excited at 514 nm and a HyD detector collected its emission between 519 nm and 570 nm. The software Fiji [24] was used to process images and to mount selected z slices, which were colorized with the “Fire” look-up table. Col-0 and PDX1.2-YFP samples were all imaged and contrasted with identical parameters; 35S-driven YFP samples, displaying a significantly higher expression, were imaged with lower laser power.

Fluorescence intensity quantifications were performed using Fiji [24] as follows: All measurements were performed on epidermal cells and only on the top-most slices of each z stack, so that signal loss due to tissue depth was negligible. On each image, a Gaussian blur (radius 0.6 pixel) was applied to reduce noise, and the average background (shot noise and detector offset) was subtracted. A line was drawn across the interface between two adjacent cells (two rhizodermal cells in roots, or a guard cell and a pavement cell in cotyledons) and the maximum intensity value encountered along the line was retained. In these thin-walled cells, the resolution of our imaging setup did not allow to discriminate the two thin cytoplasm strands of adjacent cells, which were then seen as a single line: the value measured thus gave a proxy of the average cytosolic intensity of the cell pair considered. To increase the robustness of the measurement and to reduce the uncertainty due to cytosolic fluctuations and to the presence of sub-resolution organelles, for each cell pair 5 such measurements were made, and their average value was calculated. At least two plants were analyzed for each genotype and condition, and the number of cell pairs measured in case were the following: pPDX1.2::PDX1.2-YFP L1 (n_Cotyledons -HS_ = 61; n_Cotyledons + HS_ = 63; n_Roots -HS_ = 44; n_Roots + HS_ = 37); pPDX1.2::PDX1.2-YFP L3 (n_Cotyledons -HS_ = 19; n_Cotyledons + HS_ = 30; n_Roots -HS_ = 21; n_Roots + HS_ = 24); Col-0 (n_Cotyledons -HS_ = 12; n_Cotyledons + HS_ = 8; n_Roots -HS_ = 23; n_Roots + HS_ = 41); 35S-YFP (n_Cotyledons -HS_ = 43; n_Cotyledons + HS_ = 67; n_Roots -HS_ = 54; n_Roots + HS_ = 33).

For immunochemical analysis, total protein was extracted from 8-days-old whole seedlings using the same protocol as described in [4] employing 50 mM sodium phosphate buffer, pH 7.0, containing 5 mM β-mercaptoethanol, 10 mM EDTA, 0.5% Triton X-100 [v/v], 0.1 mM PMSF, and 1% [v/v] complete plant protease inhibitor cocktail (Sigma-Aldrich) and subsequent centrifugation (16,000 *g*). The supernatant was decanted, and 30 μg of total protein (measured using the Bradford assay kit (Bio-Rad [1];) was separated on a 12% SDS-polyacrylamide gel. Immunodetection was carried out employing primary antibodies against GFP (SC8334, Santa Cruz Biotechnology, Inc.) and Actin (A0480, Sigma-Aldrich, as a loading control), both at 1:5000 dilution, the corresponding secondary antibody (peroxidase conjugated goat anti-rabbit, Bio-Rad) at 1:10000 dilution, the iBlot system (Invitrogen) and the SNAP i.d. 2.0 system (Millipore) as described by Colinas and colleagues [3]. Chemiluminescence was detected using western Bright ECL (Advansta) and captured using an ImageQuant LAS 4000 system (GE Healthcare).

### Generation of *PDX1.2* mutants by CRISPR-Cas9

Mutations were introduced into Arabidopsis *PDX1.2* through the *N*on-*H*omologous *E*nd *J*oining (NHEJ) method of RNA-guided endonuclease-mediated targeted mutagenesis with the Clustered Regularly Interspersed Short Palindromic Repeats (CRISPR)-Cas9 system [15, 16], employing the plasmids *pEN-Chimera* and *pDE-Cas9* [6] in the first instance, which were a kind gift from Prof. Holger Puchta (Karlsruhe Institute for Technology, Germany). Briefly, the specific guide RNA (sgRNA) was designed using an online tool http://crispr.mit.edu/. See Additional file 2: Table S1 for the sequence used. Two self-annealing oligonucleotides were designed with a 22 bp guide sequence targeting from + 84 in the *PDX1.2* coding sequence. For annealing, the primers were mixed in equimolar concentrations (2 μM each), denatured at 95 °C for 5 mins, cooled to ambient temperature followed by ligation (T4 DNA ligase) into *BbsI* digested gel-purified *pEN-Chimera* (1 μg) to generate *pEN-Chimera-sgPDX1.2*. The Arabidopsis U6–26 promoter and *PDX1.2* sgRNA (using 150 ng *pEN-Chimera-sgPDX1.2*) were then transferred into the destination vector *pDE-Cas9* (150 ng) by the Gateway LR recombination reaction using the LR Clonase™ II enzyme to generate *pDE-Cas9-sgPDX1.2*. The construct was introduced into *Agrobacterium tumefaciens* strain C58 and used to transform wild type Arabidopsis plants (Col-0) by the floral dip method [2]. As the *pDE-Cas9-sgPDX1.2* construct contains the *BlpR* gene, transformants were selected by resistance to phosphinothricin. Resistant plants (24 in C_1_) were allowed to self-fertilize. Plants of the C_2_ generation were checked for mutations by amplifying the region of *PDX1.2* (see Additional file 2: Table S1 for oligonucleotides used) expected to harbor Cas9 mutagenesis and identifying DNA heteroduplexes (http://www.crisprflydesign.org/t7-endo-i-assay/) by T7 endonuclease (Biolabs). Digestion of the amplicon by the T7 endonuclease indicated a Cas9 driven mutation and was verified by sequencing (Microsynth AG, Switzerland). Two independent transgenic lines with single nucleotide insertions in *PDX1.2* leading to a premature stop codon (CRISPR1, A inserted at + 89 bp; CRISPR2, C inserted at + 84 bp) were identified in this generation, however, only one (CRISPR1) could be isolated to homozygosity in the C_3_ generation and was annotated *pdx1.2–3*. Seeds harboring a single integration event were subsequently screened for loss of the Cas9 expression cassette (see Additional file 2: Table S1 for oligonucleotides used).

Mutations were also introduced into Arabidopsis *PDX1.2* with the NHEJ method of RNA-guided endonuclease-mediated targeted mutagenesis CRISPR-Cas9 system of Wang and colleagues [29], employing the plasmids pHEE2A-TRI and pHEE-401E, which were a kind gift from Prof. Qi-Jun Chen (China Agricultural University, PRC). The vectors for targeting *PDX1.2* were constructed as follows: The sequences between target 1 (+ 182 to + 204 for CRISPRA or + 436 to + 458 for CRISPRB in *PDX1.2*) and target 2 (+ 225 to + 247 for CRISPRA or + 487 to + 509 for CRISPRB in *PDX1.2*) in pHEE2A-TRI was amplified with two pairs of primers: either DT1-F0A and DT2-R0A or DT1-F0B and DT2-R0B (see Additional file 2: Table S1). The two pairs of PCR products were purified and then re-amplified with the following primers: either DT1-BsFA and DT2-BsRA or DT1-BsFB and DT2-BsRB (see Additional file 2: Table S1). The products were purified and used for cloning into the pHEE-401E plasmid using the BsaI restriction site to generate *pHEE401E-sgPDX1.2A* and *pHEE401E-sgPDX1.2B* followed by transformation into *E. coli* DH5α. Bacterial transformants were selected first on kanamycin, propagated and rechecked for kanamycin and streptomycin resistance. Colonies that were resistant only to kanamycin were chosen. Constructs were verified by sequencing (Microsynth AG, Switzerland) and introduced into *Agrobacterium tumefaciens* strain C58 and used to transform wild type Arabidopsis plants (Col-0) by the floral dip method [2]. Plant transformants were selected by resistance to hygromycin (7–8 per construct). The region of *PDX1.2* expected to harbor Cas9 mutagenesis was amplified in resistant plants of the C_1_ generation (see Additional file 2: Table S1 for oligonucleotides used) and screened by sequencing (Microsynth AG, Switzerland). Two independent transgenic lines with single nucleotide insertions in *PDX1.2* leading to a premature stop codon were identified in the C_1_ generation, allowed to self-fertilize and isolated to homozygosity. Seeds harbouring a single integration event were subsequently screened for loss of the Cas9 expression cassette. The lines isolated to homozygosity were annotated *pdx1.2–4* (CRISPRA, T inserted at bp + 187) and *pdx1.2–5* (CRISPRB, C inserted at bp + 502)*,* respectively. Potential off-targets were examined using the CRISPRdirect (https://crispr.dbcls.jp/) [20] (see Additional file 3: Table S2).

## Supplementary information

**Additional file 1: Figure S1.** Southern blot analyses of *pdx1.2–1.* Southern blot analysis of Arabidopsis genomic DNA using a probe designed to label the *BlpR* gene within the T-DNA insertion of the SAIL collection of mutants. **a** and (**b**) Autoradiographs of genomic DNA of either wild type or SAIL-640-D11 (*pdx1.2–1*) digested with either NcoI or HindIII (**a**) or EcoRI or SacI (**b**), respectively, revealing hybridization bands detected. Note that EcoRI cuts two times within the *BlpR* gene and independently of the number of T-DNA insertions gives a band of 1293 bp. (DOCX 70 kb)

**Additional file 2: Table S1.** List of oligonucleotide sequences used and their purpose within this study. List of oligonucleotide sequences used and their purpose within this study. Characters in bold correspond to primer extensions containing the sticky ends for annealing to BbsI digested pEN-Chimera. Characters in blue constitute the protospacer, i.e. the 20 nt region that precedes the PAM site targeted by the Cas9 enzyme. The BsaI restriction sites are indicated in red. Additional restriction sites for cloning are indicated in italics. (DOCX 79 kb)

**Additional file 3: Table S2.** Prediction of possible off-targets of the sgRNAs used in this study. Prediction of possible off-targets of the sgRNAs used in this study. The prediction was performed employing CRISPRdirect [20] of the *Arabidopsis thaliana* genome (version TAIR10). The search was performed with the PDX1.2 target sequences as listed. The number of sequences with a perfect match is shown and includes the on-target. In the case of CRISPRA the off-target hits are of AGI loci At1g55325 (involved in embryo patterning and cotyledon morphogenesis (Ito et al. 2011)) and At5g32690 (a pseudogene). (DOCX 79 kb)

## Data Availability

The datasets used and/or analysed during the current study are available from the corresponding author on reasonable request.

## Abbreviations

CRISPR: Clustered Regularly Interspersed Short Palindromic Repeats
HSE: heat-shock element
PLP: pyridoxal 5′-phosphate
qPCR: real-time quantitative reverse transcription PCR
TSS: transcriptional start site
YFP: Yellow fluorescent protein
