# Supplementary material for: Clarification of the dispensability of PDX1.2 for Arabidopsis viability using CRISPR/Cas9
Source: BMC Plant Biol. 2019 Nov 4;19:464. doi: 10.1186/s12870-019-2071-9 (PMC6829848; doi:10.1186/s12870-019-2071-9)
Supplement: Supplementary file 1 [file 12870_2019_2071_MOESM1_ESM.docx]

Figure S1

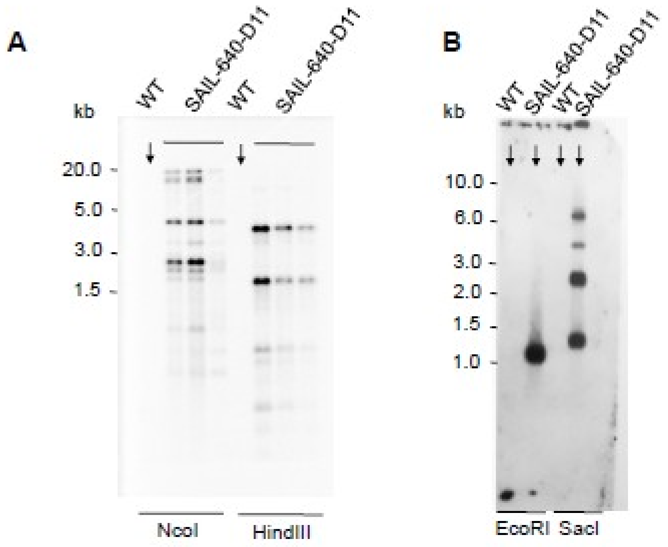
A
WT
SAIL-640-D11
WT
SAIL-640-D11
kb
20.0
5.0
3.0
1.5
NcoI
HindIII
B
WT
SAIL-640-D11
WT
SAIL-640-D11
kb
10.0
6.0
3.0
2.0
1.5
1.0
EcoRI
SacI
