# Supplementary material for: Clarification of the dispensability of PDX1.2 for Arabidopsis viability using CRISPR/Cas9
Source: BMC Plant Biol. 2019 Nov 4;19:464. doi: 10.1186/s12870-019-2071-9 (PMC6829848; doi:10.1186/s12870-019-2071-9)
Supplement: Supplementary file 2 [file 12870_2019_2071_MOESM2_ESM.docx]

Table S2. Prediction of possible off-targets of the sgRNAs used in this study.

# start	end	strand	sequence	GC	Tm	TTTT	RE_sites	hit_20mer	hit_12mer	hit_8mer	
84	106	-	CCATCCGTTCTCTGTTAAGGTCG	50.00	71.05	0		1	3	80	Sequence chosen for CRISPR1
182	204	-	CCGGCGCATGTTCTGTAATCGTC	50.00	70.29	0	NspI	1	1	20	First sequence chosen for CRISPRA
225	247	+	CGGCGGTGTTCGTCGTATGCCGG	65.00	77.92	0		1	1	32	Second sequence chosen for CRISPRA
436	458	+	ATTTGTGGTTGCCGTGATACAGG	45.00	70.04	0		1	1	23	First sequence chosen for CRISPRB
487	509	+	GCTGCTATGATTAGGATCCAAGG	45.00	70.60	0	BamHI	1	1	128	Second sequence chosen for CRISPRB

The prediction was performed employing CRISPRdirect (Naito et al., 2015) of the *Arabidopsis thaliana* genome (version TAIR10). The search was performed with the PDX1.2 target sequences as listed. The number of sequences with a perfect match is shown and includes the on-target. In the case of CRISPRA the off-target hits are of AGI loci At1g55325 (involved in embryo patterning and cotyledon morphogenesis (Ito et al 2011)) and At5g32690 (a pseudogene).

References

Ito J, Sono T, Tasaka M, Furutani M (2011) *MACCHI-BOU 2* is required for early embryo patterning and cotyledon organogenesis in Arabidopsis. Plant Cell Physiol. **52:** 539-552

Naito Y, Hino K, Bono H, Ui-Tei K (2015) CRISPRdirect: software for designing CRISPR/Cas guide RNA with reduced off-target sites. Bioinformatics **31:** 1120-1123
